# Fluorine Modified Zeolitic Imidazolate Framework Enables Long‐Life Zn–I_2_ Batteries by Suppression of Polyiodide Shuttle

**DOI:** 10.1002/anie.202513312

**Published:** 2025-09-03

**Authors:** Haoxiang Di, Yongling An, Jiarui Yang, Deyan Luan, Xiong Wen (David) Lou

**Affiliations:** ^1^ Department of Chemistry City University of Hong Kong 83 Tat Chee Avenue Kowloon Hong Kong 999077 China

**Keywords:** Polyiodide, Shuttle effect, ZIF‐8, Zn metal anode, Zn–I_2_ batteries

## Abstract

Aqueous zinc–iodine (Zn–I_2_) batteries have emerged as a promising candidate for large‐scale energy storage applications, owing to their inherent safety, cost‐effectiveness, and high specific capacity. However, their commercial implementation is severely hindered by the irreversible capacity degradation and limited cycle life, which are caused by the unavoidable iodine shuttle effect resulting from the formation of soluble I_3_^−^ species. Herein, we report the synthesis of three‐dimensional hexapod‐like fluorine‐containing zeolitic imidazolate framework (H‐F‐ZIF) nanoparticles for separator modification to effectively inhibit the iodine shuttle effect. The modified layer can interact with the I_3_^−^ species at the cathode–separator interface where the iodine shuttle occurs. Furthermore, the fluorine‐containing functional group (─CF_3_) serves as an electron acceptor, enhancing the electrostatic interaction with I_3_^−^ species, and thereby improving the polyiodide capture capacity. As a result, the Zn–I_2_ battery using H‐F‐ZIF modified separator exhibits excellent cycling durability and high capacity, retaining a capacity of 155.7 mAh g^−1^ after 12 000 cycles at 1.2 A g^−1^, and a coulombic efficiency of 86.4% after 48 h of resting.

## Graphical abstract

Hexapod‐shaped fluorine‐modified zeolitic imidazolate framework (ZIF) particles are synthesized as a functional layer on the glass fiber separator to suppress polyiodide shuttling in Zn–I_2_ batteries. The fluorine‐containing groups in the ZIF particles, served as an electron acceptor, can effectively absorb the shuttling I_3_^−^ species, enabling the stable cycling performance.

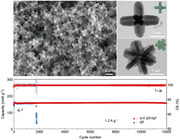

## Introduction

The growing demand for renewable energy sources is largely driven by the increasingly severe environmental pollution and the gradual depletion of fossil fuels.^[^ ^1^ ^]^ Meanwhile, the clean energy of electricity, as a substitute for fossil fuels, generated from renewable sources such as wind, solar, and tidal energy, faces a common challenge of fluctuations in output, which complicates its integration into the commercial power grid.^[^ ^2^ ^]^ Therefore, constructing large energy storage facilities is essential to store and transmit this variable electrical power to the commercial grid.^[^ ^3^, ^4^, ^5^ ^]^ In this regard, aqueous Zn metal batteries (AZMBs) present promising potential for constructing grid‐scale electrochemical energy storage systems due to their high theoretical capacity, safety, and cost‐effectiveness.^[^ ^6^, ^7^ ^]^ Specifically, Zn metal is widely used as the anode in AZMBs, offering several advantages, including a high theoretical capacity (820 mAh g^−1^),^[^ ^8^ ^]^ a relatively low redox voltage (−0.76 V versus the standard hydrogen electrode), and abundant resource availability.^[^ ^9^, ^10^ ^]^ Furthermore, the use of water‐based electrolytes containing low‐cost Zn salts contributes to the non‐flammability and cost efficiency.

Regarding cathode materials, iodine is demonstrated to be a promising candidate because of its high natural abundance of 50–60 µg L^−1^ in seawater,^[^ ^11^ ^]^ high theoretical capacity of 211 mAh g^−1^, redox potential of 0.536 V (vs SHE), and stable reversibility.^[^ ^12^ ^]^ However, the I^−^ species formed in the redox reaction during the discharge result in the formation of I_3_^−^ (I_2_ + I^−^⟶I_3_^−^).^[^ ^12^, ^13^ ^]^ The I_3_^−^ species tend to dissolve in the electrolyte and are irreversible in the following reaction, leading to the consumption of active materials and causing the capacity degradation of the cathode.^[^ ^14^, ^15^ ^]^ In addition, the dissolved polyiodide can react with the Zn anode resulting in the severe corrosion of the Zn anode.^[^ ^16^ ^]^ This phenomenon, commonly known as the shuttle effect, leads to practical challenges of Zn–I_2_ batteries including self‐discharge, irreversible capacity decay and shortened cycle life.

Various strategies have been employed to address the iodine shuttle at the cathode and promote the stability of the Zn anode. For I_2_ cathodes, the regulation of carbon materials,^[^ ^17^ ^]^ control of Prussian blue analogs^[^ ^18^ ^]^ and cellulose materials,^[^ ^19^ ^]^ and modification of binders are demonstrated to effectively mitigate the iodine shuttle.^[^ ^20^ ^]^ For Zn anodes, the construction of protective layer (ZnO,^[^ ^21^ ^]^ MXene/ZnS,^[^ ^22^ ^]^ and Al_2_O_3_^[^ ^23^ ^]^), and regulation of Zn structures^[^ ^24^, ^25^ ^]^ are reported. In addition, several previous works also demonstrate effective inhibition of the iodine shuttle effect by electrolyte engineering (hydrogel electrolytes^[^ ^26^ ^]^) and separator modification. Among these strategies, the modification of separator may be a practical way to suppress the iodine shuttle more effectively. Specifically, the separator's functions are to isolate the anode and cathode while also store the electrolyte to facilitate the ion transport. In this regard, the cathode is in close contact with the separator, forming an interface where the initial processes of iodine shuttling take place.^[^ ^27^ ^]^ Therefore, modifying the separator can directly contribute to alleviating the iodine shuttle. Recent studies have also demonstrated that the modified membranes are effective in enhancing performance of Zn–I_2_ batteries, including Janus structure modified separator,^[^ ^27^ ^]^ multifunctional zeolite membrane separator,^[^ ^28^ ^]^ ionic selective separator based on metal organic framework (MOF; UIO‐66),^[^ ^29^ ^]^ and MOF (Zn–BTC)‐multifunctional membrane.^[^ ^30^ ^]^ Among these, the MOF modification exhibits a pronounced absorption effect with polyiodides, effectively mitigating the shuttle effect. This enhancement can be attributed to the stable porous structure,^[^ ^31^ ^]^ flexible functional group modification, and abundant reaction sites in the MOF structure.^[^ ^29^, ^30^ ^]^ Therefore, modifying the separator with MOF materials is considered an effective strategy to alleviate the iodine shuttle at the cathode interface, thereby enhancing the overall performance of Zn–I_2_ batteries. Although pristine MOF materials can suppress the iodine shuttle effect through pore or channel confinement,^[^ ^30^ ^]^ such physical spatial restriction alone often proves insufficient for effectively immobilizing I_3_^−^, particularly in long‐cycle life Zn–I_2_ batteries. To address this limitation, introducing robust electrostatic confinement via functional group modification of MOFs emerges as a promising strategy to enhance I_3_^−^ suppression.^[^ ^29^ ^]^

Herein, we present a one‐step, room‐temperature wet chemical strategy to synthesize hexapod‐shaped fluorine‐modified zeolitic imidazolate framework (denoted as H‐F‐ZIF) particles as a coating layer to modify glass fiber separator (denoted as H‐F‐ZIF/GF) for iodine shuttle inhibition. Fluorine, the strongest oxidizing element in the world, exhibits a remarkable potential for the adsorption of negatively charged species.^[^ ^32^ ^]^ Thus, the high‐fluorine content functional group of –CF_3_ is selected as the electron acceptor to be introduced into the framework, thereby enhancing the trapping ability of electron‐rich I_3_^−^ through electrostatic interactions. Meanwhile, the hexapod structure of H‐F‐ZIF supports a highly porous structure within the modification layer, which enhances the efficiency for I_3_^−^ capture. Benefiting from the I_3_^−^ shuttle alleviation by H‐F‐ZIF/GF, the Zn|H‐F‐ZIF/GF|I_2_ full cells deliver excellent cycling durability and high capacity, retaining a capacity of 155.7 mAh g^−1^ after 12 000 cycles at 1.2 A g^−1^, and a high coulombic efficiency (CE) of 86.4 % after 48 h of resting.

## Results and Discussion

**Figure 1:**
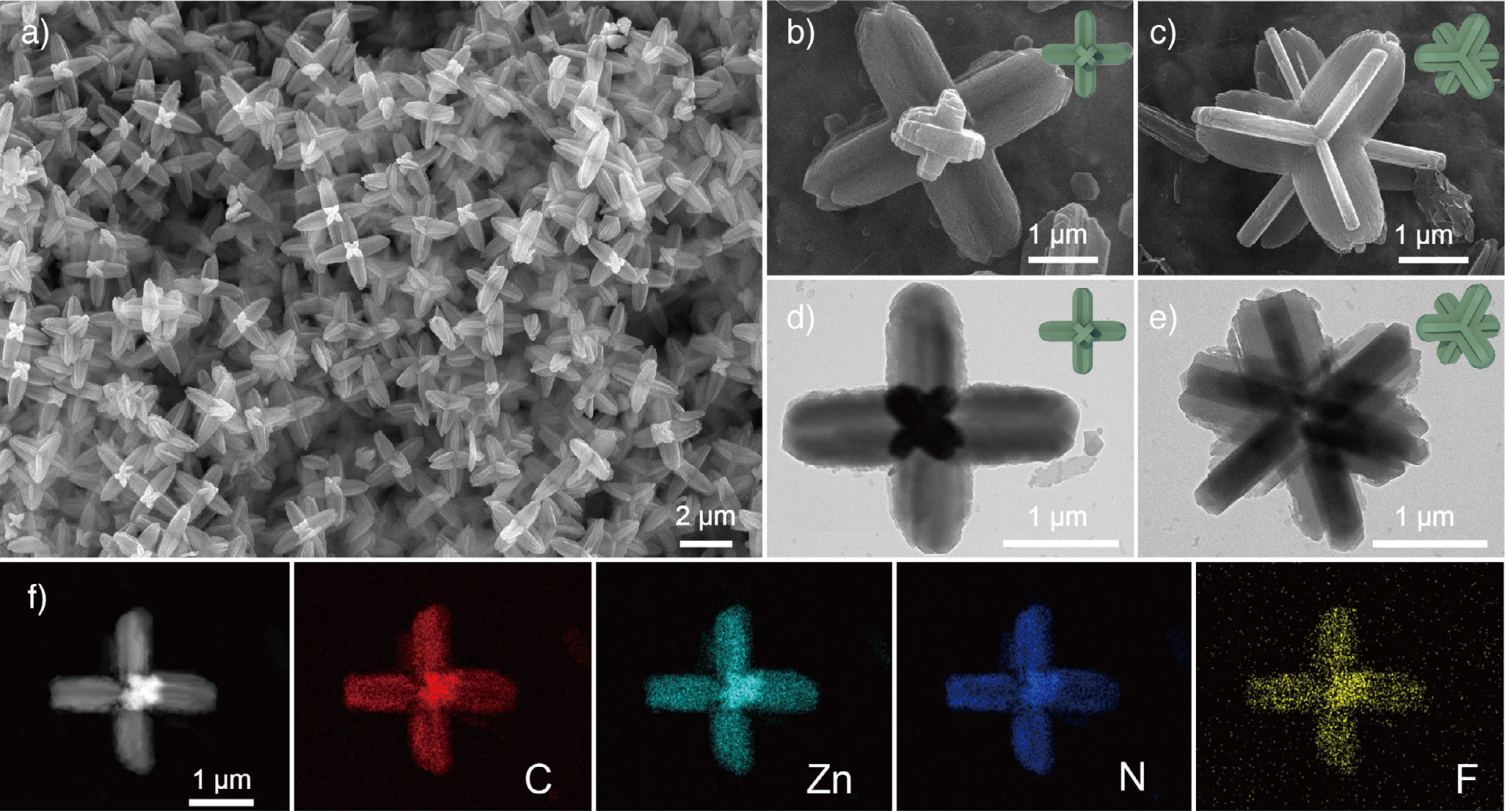
a) FESEM image of H‐F‐ZIF. b) and c) FESEM and d) and e) TEM images of H‐F‐ZIF particles in different orientations with the insets showing the schematic models. f) Elemental mapping images of H‐F‐ZIF.

The H‐F‐ZIF particles are synthesized through a simple one‐step solution method by mixing Zn(CH_3_COO)_2_ aqueous solution and 2‐methylimidazole/trifluoroacetic acid (TFA) aqueous solution. As revealed by field‐emission scanning electron microscopy (FESEM) images, the hexapod structure comprises three rods oriented orthogonally in three‐dimensional space (Figure 1a–c). The elaborated morphology is shown in the high magnification FESEM (Figure 1b,c) and transmission electron microscopy (TEM) images (Figure 1d,e), and the schematic models in different orientations are also displayed in the relevant figures. Every rod is assembled from two rectangular strips like a cruciform prism. Three rods intersect orthogonally at a shared central point, forming H‐F‐ZIF particles. The hexapod architecture can effectively increase the contact surface area with the electrolyte,^[^ ^33^ ^]^ which enhances the probability of I_3_^−^ capture. To better understand the structural features of H‐F‐ZIF, three‐dimensional models of H‐F‐ZIF in different rotation angles are shown in Figure S1. The morphology evolution of H‐F‐ZIF at different reaction stages is observed by terminating the reaction at 5, 30, 60, and 180 min, as displayed in Figure S2. The highest product yield is obtained after the reaction time of 180 min. Six tentacle‐like humps in orthogonal directions could be observed in 5 min (Figure S2a), which reveals a hexagonal growth orientation of H‐F‐ZIF during the initial nucleation stage. Then, the orthorhombic strip morphology gradually develops on each rod concomitant with particle volume expansion in 30 to 60 min (Figures S2b,c,). Subsequent observation reveals the volume expansion and the pod elongation in 180 min (Figure S2d). Energy‐dispersive X‐ray (EDX) spectroscopy elemental mapping is displayed to uncover the element distribution as shown in Figure 1f. C, N, F, and Zn elements are uniformly allocated over the particles, indicating the successful introduction of F element. The EDX spectrum further confirms the successful inclusion of fluorine in H‐F‐ZIF (Figure S3).

**Figure 2:**
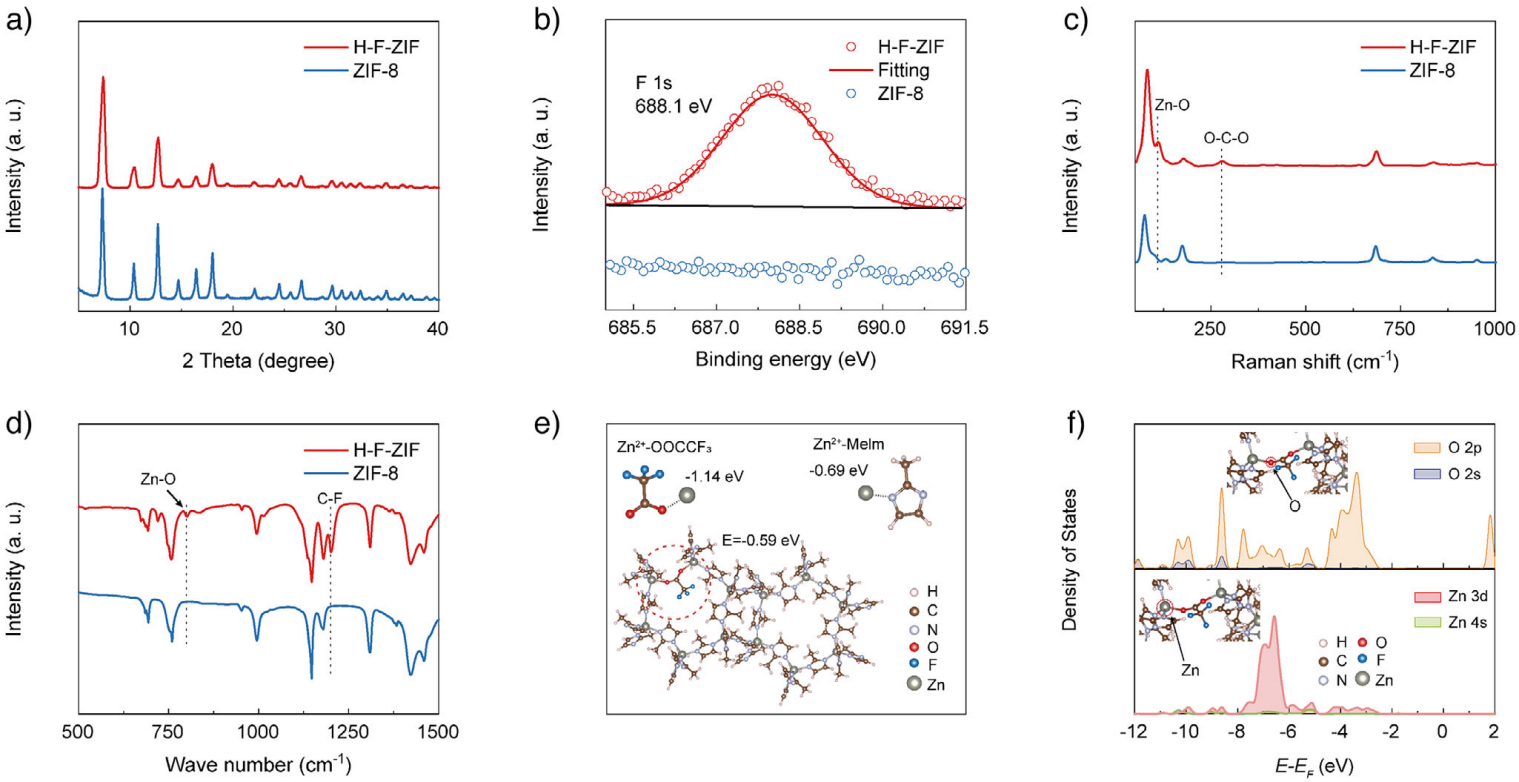
a) XRD patterns, b) F 1s XPS spectra, c) Raman, and d) FTIR spectra of ZIF‐8 and H‐F‐ZIF. e) DFT calculated model of H‐F‐ZIF. f) Density of states analysis of Zn and O in H‐F‐ZIF.

X‐ray diffraction patterns (XRD) show that the H‐F‐ZIF remains the same crystal structure with ZIF‐8,^[^ ^34^ ^]^ which indicates that the introduction of ─CF_3_ does not destruct the intrinsic crystal structure of ZIF‐8 (Figure 2a). X‐ray photoelectron spectroscopy (XPS) is performed to prove the introduction of fluorine (Figures S4a,b). As shown in Figure 2b, an obvious peak at 688.1 eV which is related to the C─F species in ─CF_3_, is observed in H‐F‐ZIF,^[^ ^35^ ^]^ while the pristine ZIF‐8 shows no peak at the same energy range, confirming the successful incorporation of fluorine.

Then, Raman spectroscopy is used to study the chemical structure of H‐F‐ZIF. As shown in Figure 2c, the Raman shifts at 110 and 279 cm^−1^ correspond to Zn─O bend vibration and O─C─O deform vibration.^[^ ^36^ ^]^ Fourier transform infrared (FTIR) spectroscopy is utilized to further demonstrate the chemical interaction of Zn─O (Figure 2d). In comparison with the FTIR spectrum of pristine ZIF‐8, the peak appeared at ∼800 cm^−1^ responds to Zn─O stretching vibration in H‐F‐ZIF,^[^ ^37^ ^]^ while the additional peak between 1000–1300 cm^−1^ is attributed to the C─F band vibration.^[^ ^38^ ^]^ The Raman and FTIR results suggest that the TFA molecule coordinates in the framework with Zn─O chemical interaction. To rule out the possibility that the Zn─O interaction might form even in the physical adsorption between TFA and ZIF‐8, the Raman spectrum of ZIF‐8 soaked in TFA is obtained (Figure S5). Pristine ZIF‐8 is immersed in an aqueous TFA solution for 4 h and dried in a vacuum oven overnight before the Raman test. Compared with the pristine ZIF‐8, the soaked sample shows a Raman shift at ∼279 cm^−1^, corresponding to O─C─O in TFA, confirming the adsorption of TFA. However, the Zn─O shift is not found at around 110 cm^−1^ compared with the H‐F‐ZIF, demonstrating that the physical absorption will not form Zn─O interaction. As a result, the TFA likely coordinates with the framework through the Zn─O chemical interaction rather than physical absorption, which may enhance the combination stability of ─CF_3_ in H‐F‐ZIF.

Density functional theory (DFT) study is then performed to reveal the coordination tendency between the TFA and ZIF framework, as shown in Figure 2e. The free energy analysis reveals that the Zn^2+^ cation forms a more stable coordination with the TFA (−1.14 eV) than with 2‐Methylimidazole (denoted as Melm, −0.69 eV), indicating a preferential coordination between TFA and Zn^2+^ during the nucleation process. Furthermore, the formation energy of H‐F‐ZIF is −0.59 eV, suggesting a thermodynamically stable coordination of TFA in H‐F‐ZIF. The simulated H‐F‐ZIF unit is shown in Figure 2e and the H‐F‐ZIF model containing four‐unit cells is shown in Figure S6, revealing that the TFA displaces a Melm ligand and coordinates with the Zn center in the framework. The partial density of states (PDOS) analysis is performed based on the above H‐F‐ZIF model. As illustrated in Figure 2f, the PDOS of O 2p and O 2s is displayed in the upper section, and the PDOS of Zn 3d, Zn 4s is presented in the lower section. Significant overlap on electron states between Zn 3d and O 2p is observed in the energy range of −2.7 to −4.5 eV, −6.3 to −7.7 eV, −8.5 to −9.0 eV, and −9.8 to −10.2 eV, suggesting strong hybridization between these orbitals, which facilitates the chemical interaction between Zn and O. The total DOS of H‐F‐ZIF is shown in Figure S7. The structural analogue ZIF‐8, which lacks ─CF_3_ functional groups, is employed as a control group to elucidate the specific role of ─CF_3_ of H‐F‐ZIF in subsequent tests.

**Figure 3:**
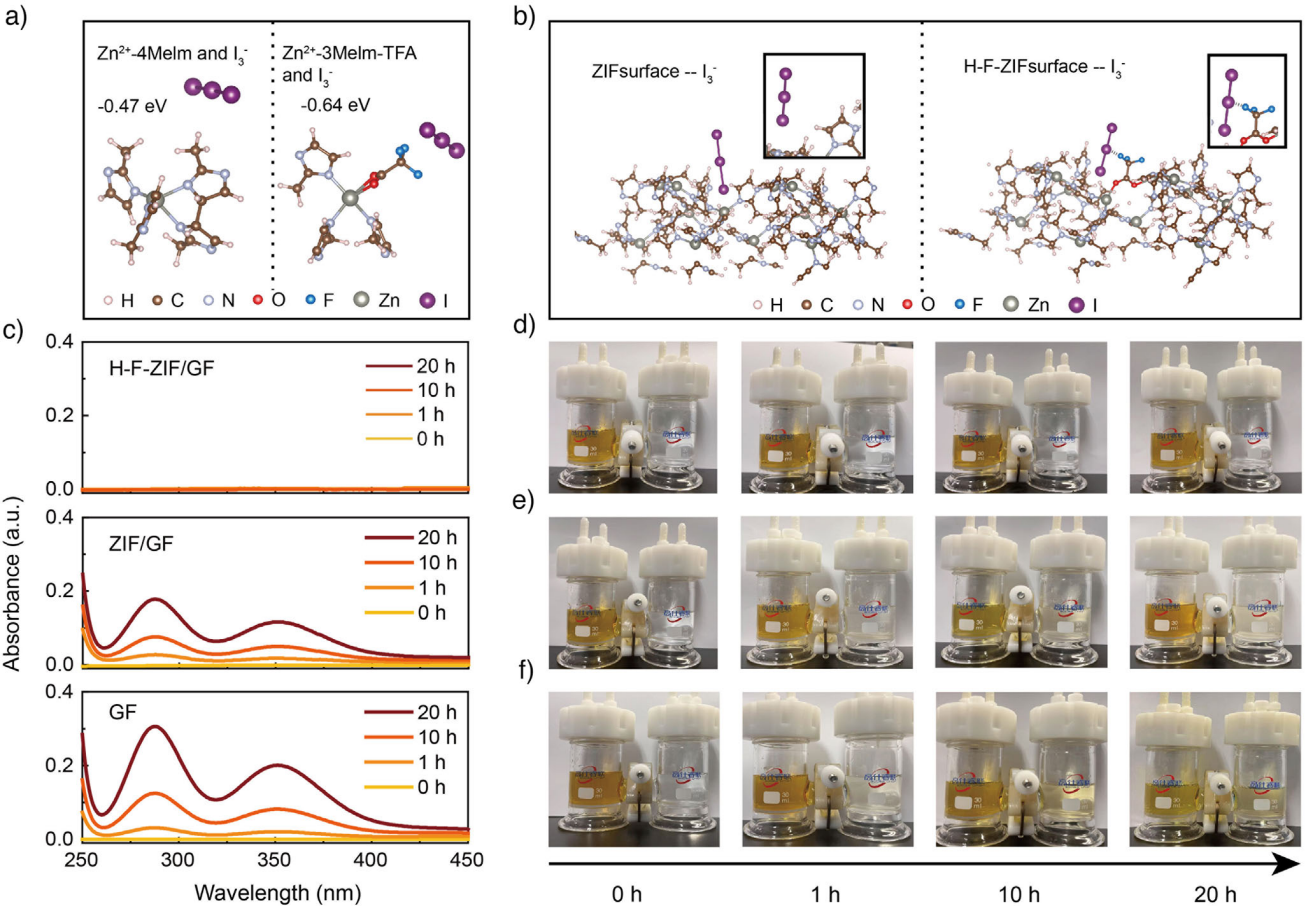
a) Calculation models of the interaction between the I_3_^−^ ion and Zn^2+^‐4Melm unit (left), and the I_3_^−^ ion and Zn^2+^‐3Melm‐TFA unit (right). b) DFT calculated absorption models of I_3_^−^ on the surface of ZIF and H‐F‐ZIF. c) UV–vis curves representing the change in I_3_^−^ concentration over time in the right chambers of H‐type cells using H‐F‐ZIF/GF, ZIF/GF, and GF. Optical images of the permeation experiments of I_3_^−^ in the H‐type cell assembled with d) H‐F‐ZIF/GF, e) ZIF/GF, and f) GF.

DFT calculations are conducted to investigate the I_3_^−^ absorption on H‐F‐ZIF and ZIF‐8. First, the interaction between the I_3_^−^ and the coordination unit is simulated. As shown in Figure 3a, the free energy between I_3_^−^ and the Zn^2+^‐Melm unit in ZIF‐8 is −0.47 eV, and it is −0.64 eV between I_3_^−^ and the Zn^2+^‐Melm‐TFA in H‐F‐ZIF, which demonstrates that the –CF_3_ group can strengthen the absorption of I_3_^−^. Since the electrolyte is initially in contact with the material surface, we then calculate the absorption model of I_3_^−^ on the surfaces of ZIF‐8 and H‐F‐ZIF, the surface models are shown in Figures S8 and S9. As shown in Figure 3b (and Figures S10, S11), the adsorption energy of I_3_^−^ is −0.56 eV on ZIF‐8 and −0.95 eV on H‐F‐ZIF, indicating that the electron‐withdrawing –CF_3_ group can effectively enhance the I_3_^−^absorption.

The H‐F‐ZIF/GF fabricated by the air spray gun method is prepared for iodine absorption assessment. As shown in Figure S12, the XRD pattern of H‐F‐ZIF/GF exhibits characteristic peaks of H‐F‐ZIF, demonstrating its successful decoration on the GF separator. The FESEM (Figures S13 and S14a,b) and cross‐sectional FESEM images (Figures S14c,d) of GF and H‐F‐ZIF/GF display that the surface of the pristine GF is chaotically covered with many microfibers. In contrast, after the H‐F‐ZIF modification, a dense layer is covered on the separator, and the thickness of the H‐F‐ZIF layer is about 19 µm.

To visually compare the polyiodide species shuttle through different separators, H‐shape cells are assembled with the pristine GF, ZIF‐8 modified glass fiber (denoted as ZIF‐8/GF), and H‐F‐ZIF/GF separators, where the left chamber contains brown triiodide solution (Figure 3d–f). The coated side of H‐F‐ZIF/GF and ZIF‐8/GF is oriented toward the left chamber containing triiodide solution. With the normal GF separator (Figure 3f), the colorless solution in the right chamber turns to light yellow after 1 h, which indicates the shuttle of I_3_^−^ through the GF. Then, the right‐side solution gradually becomes into brown and approaches the diffusion equilibrium in about 20 h. In the ZIF‐8/GF separator group (Figure 3e), the polyiodide shuttle is partially suppressed, but the right chamber also becomes brown after 20 h. In contrast, with the H‐F‐ZIF/GF separator, the right chamber maintains colorless during the 20 h period (Figure 3d), which demonstrates efficient inhibition of polyiodide shuttle. Furthermore, the quantitative analysis of the I_3_^−^ concentration in the right chamber is conducted using UV–vis absorption spectroscopy. The standard curves for I_3_^−^ concentration and UV–vis absorption intensity are shown in Figure S15, where the characteristic peak of I_3_^−^ locates at about 288 nm.^[^ ^16^ ^]^ As shown in Figure 3c, in the H‐shape container with the pristine GF separator, the concentration of I_3_^−^ in the right chamber reaches 3.07 × 10^−2^ mM in the first 10 h, and then increases to 6.58 × 10^−2^ mM in 20 h. For the ZIF‐8/GF, the concentration reaches about 2.09 × 10^−2^ mM in 10 h and 4.08 × 10^−2^ mM in 20 h. Notably, no I_3_^−^ is detected in the H‐F‐ZIF/GF group even after 20 h, demonstrating that the I_3_^−^ shuttle is significantly alleviated by the H‐F‐ZIF. Moreover, polyiodide permeation experiments are also conducted at higher I_3_^−^concentrations. The H‐cell with GF separator shows obvious polyiodide shuttling in 0.5 h (Figures S16a,c), while the H‐F‐ZIF/GF effectively suppresses shuttling with no detectable I_3_^−^ in 12 h (Figures S16b,d).

The shuttling polyiodide species can also induce corrosion of the Zn anode. To evaluate the corrosion suppression ability of modified separator, fresh Zn foil is soaked in the left chamber of the H‐type cell (Figure S17) for 72 h using either GF or H‐F‐ZIF/GF separator. The FESEM image (Figure S18a) reveals that the Zn foil immersed in the pristine GF assembled cell exhibits a rough and disordered surface morphology with numerous bumps, indicating the polyiodide shuttles through the GF separator and ultimately leading to severe Zn foil corrosion. In contrast, the Zn foil from the H‐F‐ZIF/GF cell maintains a smooth surface morphology, demonstrating that the H‐F‐ZIF/GF effectively suppresses I_3_^−^ shuttle and inhibits the I_3_^−^ corrosion (Figure S18b).

To quantitatively investigate the polyiodide shuttle effect during the charge–discharge cycles, ex situ UV–vis spectroscopy is performed using an H‐type cell assembled with either a pristine GF separator or an H‐F‐ZIF/GF separator. The solution in the left chamber (anode side) is sampled at different charge and discharge stages for I_3_^−^ concentration analysis. As shown in Figures S19a and S20, when using the pristine GF separator, I_3_^−^ first appears upon charging to 1.25 V, and the concentration is 1.77 × 10^−2^ mM. Then, it continually accumulates to 4.55 × 10^−2^ mM when discharged to 0.5 V. With H‐F‐ZIF/GF, no I_3_^−^ absorption peak is detected, indicating its effective inhibition of polyiodide shuttle during cycling (Figure S19b).

**Figure 4:**
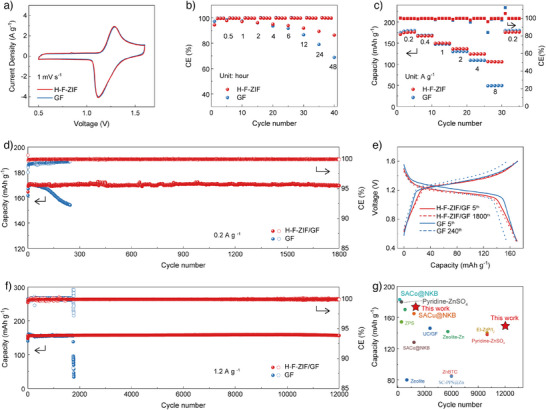
a) CV curves at a scan rate of 1 mV s^−1^, b) CE during different aging periods, c) rate performance, d) cycling performance at 0.2 A g^−1^ and e) corresponding charge–discharge voltage profiles at 0.2 A g^−1^, and f) cycling performance at 1.2 A g^−1^ of the Zn–I_2_ cells with GF and H‐F‐ZIF/GF separators. g) Comparison of electrochemical performance for Zn–I_2_ batteries in this work with some reported Zn–I_2_ batteries.

Zn–I_2_ full batteries are assembled to assess the practical application of H‐F‐ZIF/GF. Activated carbon (AC) is used as the host for loading iodine. FESEM images of the AC cathode without iodine loading are shown in Figure S21. The cross‐sectional elemental mapping images and XRD patterns of the AC cathode with iodine loading are shown in Figures S22 and S23, respectively. The specific capacities are calculated based on the mass of iodine in the cathode. In addition, the AC cathode without iodine is tested at a current density of 1.2 A g^−1^. The result verifies that AC can provide very low capacity and no voltage platform (Figure S24). Thus, iodine is confirmed to be the primary capacity source in Zn–I_2_ batteries. The optimal H‐F‐ZIF loading on the GF is determined by balancing polyiodide shuttle suppression and electrochemical performance. Cyclic voltammetry (CV) curves of GF and H‐F‐ZIF/GF batteries are shown in Figure 4a. It can be analyzed that both cells exhibit analogical curves with a pair of iodine redox peaks, which indicates that the H‐F‐ZIF modification has no effect on the electrochemical reaction. Furthermore, the similar CV curve areas observed for both H‐F‐ZIF/GF and GF based batteries demonstrate that the coating layer has negligible influence on the specific capacity. Subsequently, the long‐term resting test is performed, where the fully charged cells are rested for different durations, after which their discharge capacities are measured to determine the CE. As shown in Figure 4b, the Zn–I_2_ cell with GF separator suffers a slight capacity loss of 3.94% and 8.30% after 2 and 6 h of resting, respectively. However, the self‐discharge becomes serious as the aging time increases, and the CE is 79.05% and 68.83% after 24 and 48 h resting, respectively (Figures S25a and c). The serious capacity loss reveals that the active material uncontrollably shuttles into the electrolyte. While the cell with the H‐F‐ZIF/GF separator initially loses 3.75% and 6.02% of capacity for 2 and 6 h of resting, respectively which is similar to the cell with GF. Then, the capacity retainment of the cell with H‐F‐ZIF/GF researches 89.41% and 86.42% after 24 and 48 h resting (Figures S25b and d), which are obviously better than the cell with GF. Clearly, the H‐F‐ZIF/GF can effectively inhibit the shuttle of iodine species and alleviate the self‐discharge of Zn–I_2_ cells. The rate performance under different current densities from 0.2 to 8 A g^−1^ is displayed in Figure 4c (and Figure S26). The Zn–I_2_ cell with H‐F‐ZIF/GF exhibits capacities of 176.4, 167.2, 149.7, 136.5, 124.2, and 105.8 mAh g^−1^ at current densities of 0.2, 0.4, 1, 2, 4, and 8 mA g^−1^, respectively. The capacity recovers to 176.4 mAh g^−1^ when the current density reverses to 0.2 A g^−1^. In contrast, the Zn–I_2_ full cell with GF separator only shows a similar capacity with H‐F‐ZIF/GF under a low current density, but sharply drops to 109.1, and 49.6 mAh g^−1^ under the current densities of 4 and 8 mA g^−1^, respectively. Therefore, the modified H‐F‐ZIF/GF separator notably enhances the rate performance. The electrochemical impedance spectroscopy results demonstrate that the cycled H‐F‐ZIF/GF cell exhibits significantly reduced ohmic resistance (*R_0_* = 2.8 Ω) and charge transfer resistance (*R_ct_* = 97.9 Ω) compared to the GF cell (*R_0_* = 10.8 Ω, *R_ct_* = 229.6 Ω), indicating enhanced ionic conductivity of the former (Figure S27). This improved charge transfer of the cycled cell originates from the H‐F‐ZIF/GF's effective suppression of I_3_^−^ shuttle‐induced Zn corrosion and side reactions.^[^ ^39^, ^40^ ^]^ The elevated discharge voltage of the cycled H‐F‐ZIF/GF cell also verifies the improved electrochemical kinetics (Figure S28). The galvanostatic cycling test at a current density of 0.2 A g^−1^ is shown in Figure 4d, and the corresponding charge/discharge voltage curves are shown in Figure 4e. After several activation cycles, the capacity of the Zn–I_2_ battery with GF separator reaches 170.4 mAh g^−1^. Nevertheless, the capacity begins to decline after 100 cycles. After 245 cycles, the capacity decays to 154.1 mAh g^−1^. Remarkably, the Zn–I_2_ cell with the H‐F‐ZIF/GF separator reaches a maximum capacity of 170.9 mAh g^−1^, and it only decays to 169.3 mAh g^−1^ after 1700 cycles. The relatively stable cycling performance under the low current density can be attributed to the effective inhibition of I_3_^−^ shuttle by H‐F‐ZIF. In addition, the results also suggest that the trapped I_3_^−^ is not permanently adsorbed but rather participates reversibly in the electrochemical reactions. If irreversible adsorption occurred, the capacity would inevitably decline over time, which is not observed in the measurement. The excellent cycling performance is further demonstrated when the current density increases to 1.2 A g^−1^, as shown in Figure 4f. The cell with H‐F‐ZIF/GF exhibits a maximum capacity of 158.7 mAh g^−1^. Even after 12 000 cycles, it still retains a capacity of 155.7 mAh g^−1^. The voltage profiles of the Zn–I_2_ cell with H‐F‐ZIF/GF after 10 000 cycles are shown in Figure S29. In contrast, the Zn–I_2_ cell with pristine GF separator shows a close initial capacity, but it suffers a sharp decline of capacity only after 1770 cycles. The XRD pattern of cycled H‐F‐ZIF/GF (Figure S30) retains characteristic peaks of pristine H‐F‐ZIF (Figure 2a), demonstrating its structural stability during cycling. The cycled Zn anode is further characterized to evaluate the suppression of the iodide shuttle by H‐F‐ZIF/GF. FESEM images (Figure S31) reveal that the Zn anode in the H‐F‐ZIF/GF cell maintains a smoother surface with fewer pits, suggesting effective mitigation of triiodide corrosion.^[^ ^30^ ^]^ This improved suppression is further supported by XRD (Figure S32) and EDX (Figure S33) analyses, which show reduced surface degradation and suppressed iodide byproduct formation, confirming the inhibition of the shuttle effect by H‐F‐ZIF/GF.^[^ ^41^ ^]^ In addition, the Zn–I_2_ cell with H‐F‐ZIF/GF separator also displays a respectable cycling performance and a competitive specific capacity compared with some representative reports,^[^ ^4^, ^16^, ^28^, ^29^, ^30^, ^42^, ^43^, ^44^, ^45^ ^]^ as shown in Figure 4g. The high reversibility and long life of the Zn–I_2_ cell can be attributed to the inhibition of I_3_^−^ shuttle by the H‐F‐ZIF/GF layer.

To demonstrate the practical application of H‐F‐ZIF/GF, we assemble Zn–I_2_ batteries with a high iodine loading of 10 mg cm^−2^. The H‐F‐ZIF/GF‐based battery maintains excellent performance, delivering a high CE of 99.8% and a specific capacity of 146.6 mAh g^−1^ after 500 cycles (Figure S34). Furthermore, as shown in Figure S35, a Zn–I_2_ pouch cell (5 cm × 5 cm) is also assembled exhibiting stable cycling performance, retaining a capacity of 172.1 mAh g^−1^ with a CE of 99.5% after 80 cycles. In addition, the H‐F‐ZIF/GF separator also demonstrates excellent performance in Zn//Zn symmetric and Zn//Cu asymmetric cells (Figures S36a,b), showing superior reversibility and cycling stability. CV curves (Figure S36c) reveal a higher onset potential for Zn plating with H‐F‐ZIF/GF, indicating reduced polarization and enhanced reaction kinetics, suggesting its potential application in Zn‐based battery systems.

## Conclusion

In summary, an H‐F‐ZIF modification layer on separator is reported to achieve highly reversible Zn–I_2_ batteries by efficient iodine shuttle suppression. The H‐F‐ZIF is elaborately designed and synthesized through a facial one‐step wet chemical strategy at room temperature. The ─CF_3_ functional group serves as a strong electron acceptor, improving I_3_^−^ capture via enhanced electrostatic interactions. As a result, the Zn|H‐F‐ZIF/GF|I_2_ full cell displays excellent cycling stability of 12 000 cycles with a capacity of 155.7 mAh g^−1^ at 1.2 A g^−1^, and about 1800 cycles with a capacity of 169.3 mAh g^−1^ at 0.2 A g^−1^. In addition, a high CE of 86.42% is also achieved by the H‐F‐ZIF/GF even after 48 h of resting. This work presents a simple and reliable strategy for constructing H‐F‐ZIF particles to alleviate I_3_^−^ shuttling in Zn–I_2_ batteries, thus offering a new design approach for highly reversible Zn–I_2_ batteries.

## Conflict of Interests

The authors declare no conflict of interest.

## Supporting information



Supplementary Information

## Data Availability

The data that support the findings of this study are available from the corresponding author upon reasonable request.
