# Supplementary material for: Fluorine Modified Zeolitic Imidazolate Framework Enables Long‐Life Zn–I2 Batteries by Suppression of Polyiodide Shuttle
Source: Angew Chem Int Ed Engl. 2025 Sep 3;64(43):e202513312. doi: 10.1002/anie.202513312 (PMC12535394; doi:10.1002/anie.202513312)
Supplement: Supplementary file 1 — Supplementary Information [file ANIE-64-e202513312-s001.pdf]

Supporting Information for

Fluorine Modified Zeolitic Imidazolate Framework Enables Long-Life Zn-I_2 Batteries by Suppression of Polyiodide Shuttle

*Haoxiang Di, Yongling An, Jiarui Yang, Deyan Luan, Xiong Wen (David) Lou**

[*] H. X. Di, Dr. Y. L. An, Dr. J. R. Yang, Dr. D. Y. Luan, Prof. X. W. Lou

Department of Chemistry, City University of Hong Kong, 83 Tat Chee Avenue, Kowloon, 999077, Hong Kong, China   Email: david.lou@cityu.edu.hk

Experimental details

Synthesis of the hexapod-shaped fluorine modified ZIF (H-F-ZIF). In a typical preparation, 600 mg of $Zn(CH_3COO)_2 \cdot 2H_2O$ is dissolved in 5 mL of deionized water to form solution A. 1.12 g of 2-methylimidazole is dissolved in 5 mL of deionized water, then 10 μL of Trifluoroacetic acid (TFA) is slowly dropped into the solution under ice-bath in 30 minutes to form solution B. Due to its high volatility and acidity, TFA handling should be conducted in a fume hood with an ice bath. Solution B is then being stirred until recover to room temperature. Then, solution A is poured into solution B and the mixture is stirred for 30 seconds. Afterwards, the solution is left undisturbed at room temperature for 180 min. The resulting productions are washed with ethanol and collected by centrifugation, then dried at 60 °C for overnight.

Fabrication of H-F-ZIF/GF separator. H-F-ZIF and polyvinylidene fluoride (PVDF) are mixed in a weight ratio of 9:1 with N-methylpyrrolidone (NMP) as the dispersant. Stirring and ultrasonically dispersing to obtain the even slurry. The slurry is sprayed on the glass fiber (GF) separator on one side by the electric spray gun. The obtained separator is dried at 80 °C for 12 h in vacuum oven.

Synthesis of the ZIF-8. In a typical synthesis, 300 mg of $Zn(CH_3COO)_2 \cdot 2H_2O$ is dissolved in 5 mL of deionized water to form solution A, and 1.12 g of 2-methylimidazole is dissolved in 5 mL of deionized water to form solution B. Then, solution A is added into solution B under stirring. Afterwards, the solution is left undisturbed at room temperature for 4 h. The resulting productions are washed with ethanol and collected by centrifugation, then dried at 60 °C for overnight.

Preparation of iodine cathode. The active carbon (YP80F), conductivity carbon (SuperP), and polytetrafluoroethylene binder emulsion are mixed in a mass ratio of 8:1:1. The mixture is uniformly stirred to be pressed onto the titanium mesh, and then dried in a vacuum oven at a temperature of 60 °C

overnight. The catholyte prepared by dissolving 0.1 M I_2 and 0.5 M ZnI_2 into DI water is dropped on the dried cathode to provide iodine active materials. The mass loading for iodine species is about 4 mg cm^{-2}.

Material characterizations. The morphology and structure of the samples are characterized by scanning electron microscopy (SEM; QUATTRO-S) and transmission electron microscopy (TEM; JEOL JEM-F200). The compositions are analyzed by energy-dispersive X-ray spectroscopy (EDX) attached to the TEM instruments. The crystal phases of the samples are analyzed by X-ray diffraction (XRD) on a Bruker D2 Phaser X-ray diffractometer with Ni filtered Cu Kα radiation (λ = 1.5406 Å) at a voltage of 30 kV and a current of 10 mA. The chemical compositions are calculated by the X-ray photoelectron spectroscopy (XPS, Thermo Fischer, ESCALAB 250Xi). The binding energy is calibrated by C 1s peak (284.8 eV). The Fourier transform infrared spectrum (FTIR) is performed by the Perkin Elmer Spectrum 100 in the range of 400-3000 cm^{-1} based on the KBr pellet method. The test of I_3^- concentration is performed by SHIMASZU UV-2600i. The specific surface area is tested from the nitrogen adsorption isotherms based on the Brunauer-Emmett-Teller (BET) model by the Micrometrics ASAP 2020.

Electrochemical measurements. Electrochemical tests are carried out using CR2016 coin cells. 100 μL of 2 mol L^{-1} $ZnSO_4$ aqueous solution is utilized as the electrolyte for all cells. A zinc foil of 16 mm in diameter, 20 μm in thickness is employed as the Zn anode, and H-F-ZIF/GF or GF are used as separators. The coated side of H-F-ZIF/GF is oriented toward the cathode during cell assembly. The galvanostatic charge-discharge tests are conducted by the Neware battery test system (MIHW-200-160CH-B), and the cyclic voltage tests (CV) are conducted using the electrochemical work station (CHI760E). The voltage range of 0.5-1.6 V is set for all the tests.

Computational details. Vienna ab initio simulation package (VASP) is used to perform the first-principle calculation based on the density functional theory.[1-3] The projector augment wave (PAW) is selected to describe the ion-electron interaction.[4, 5] The exchange correlation energy functional in generalized gradient approximation (GGA) is modeled by the scheme of Perdew-Burke-Ernzerhof (PBE).[6] The cutoff energy for the plane-wave is set to 450 eV. A convergence threshold of 1×10^{-5} eV in energy for self-consistent field (SCF) electronic iteration and 0.02 eV $Å^{-1}$ in force for geometry optimizations are set for accuracy during the simulations. In addition, the unit structures are relaxed within the 3×3×3 Monkhorst-Pack K-point grid, and the periodic structures are optimized in Gamma-point K grid. A 15 Å vacuum space along the Z axis is placed to avoid the interaction among the slab.

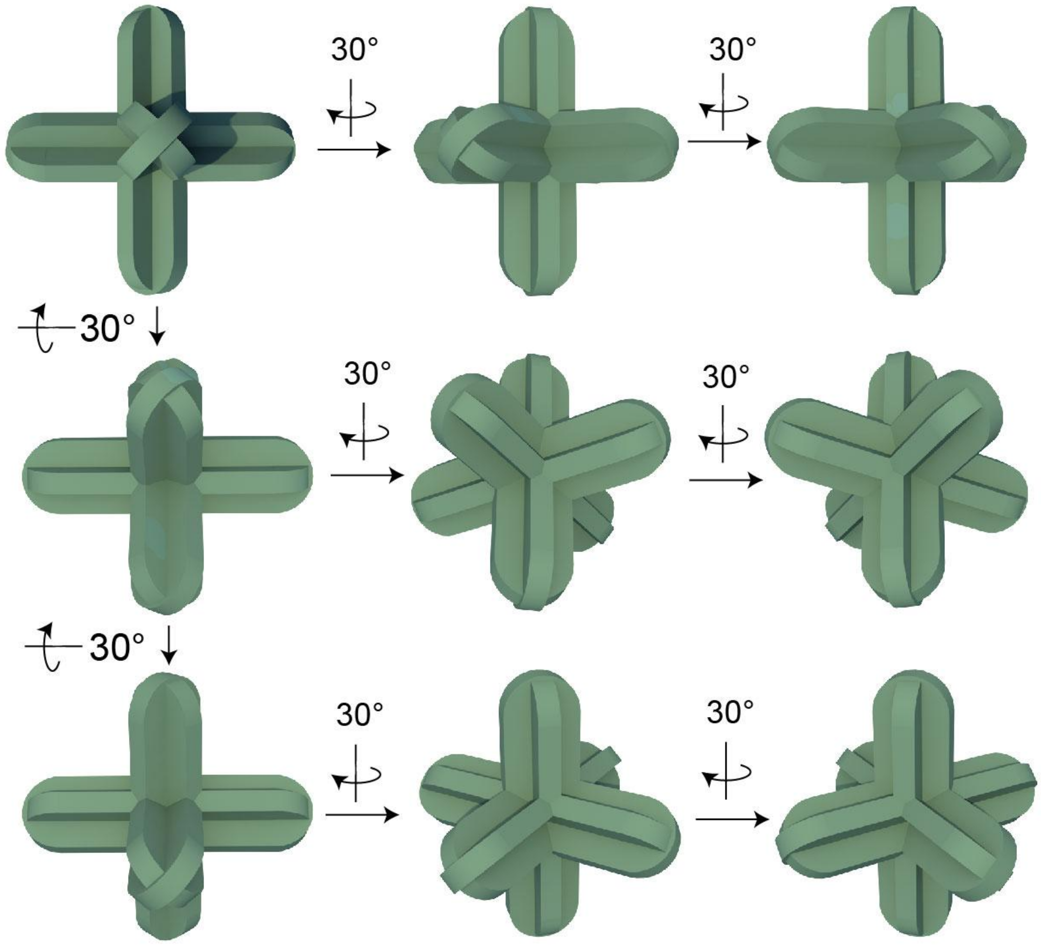

Figure S1. Schematic models of the H-F-ZIF particle in different orientations.

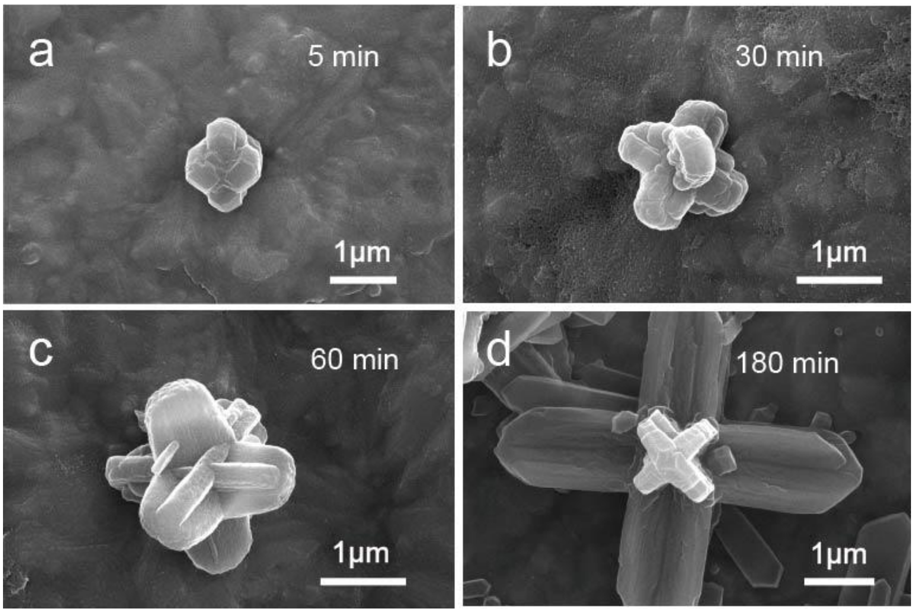

Figure S2. FESEM images of the samples obtained at a) 5 min, b) 15min, c) 60 min, and d) 180 min of the reaction process.

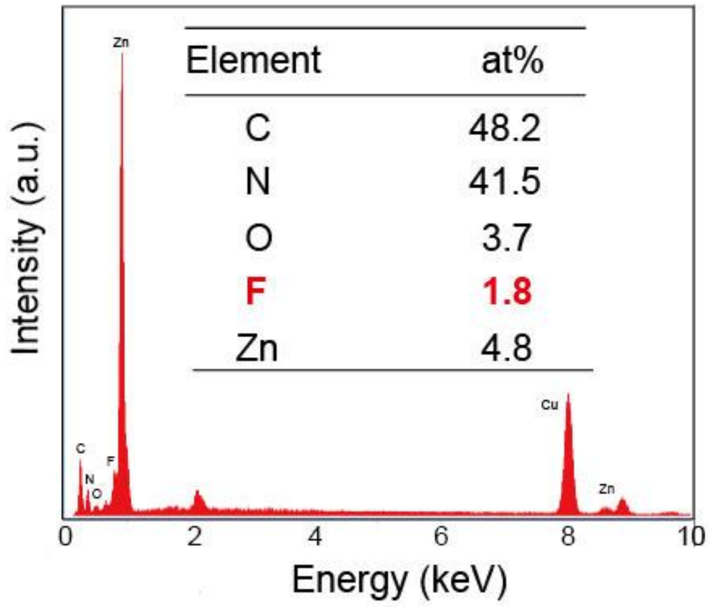

Figure S3. EDX spectrum of H-F-ZIF.

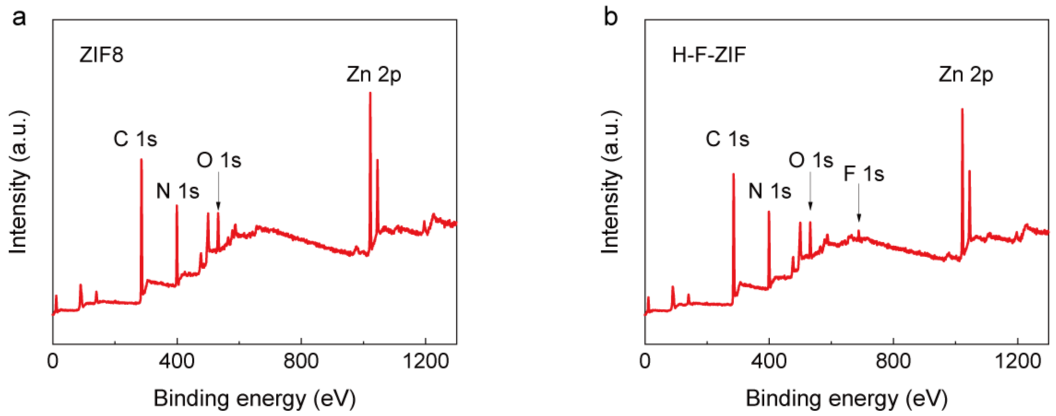

Figure S4. XPS survey spectra of a) ZIF-8 and b) H-F-ZIF.

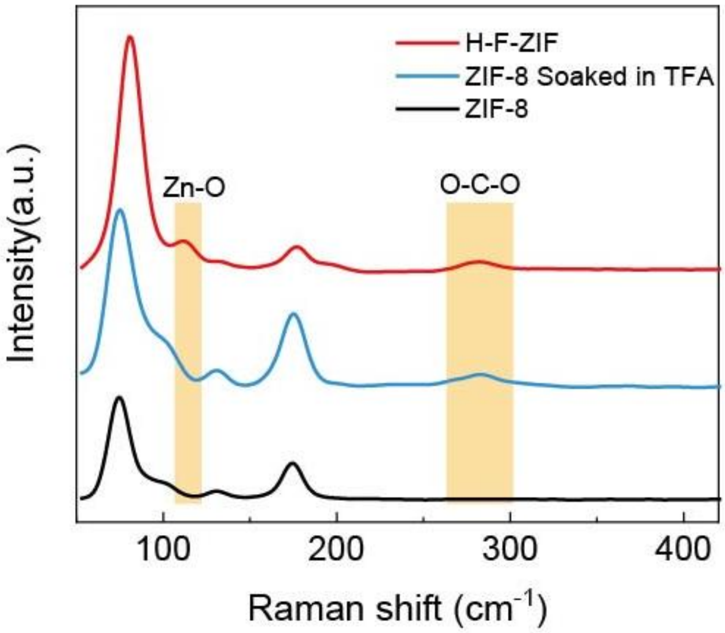

Figure S5. Raman spectra of ZIF-8 and ZIF-8 soaked in the TFA solution and H-F-ZIF.

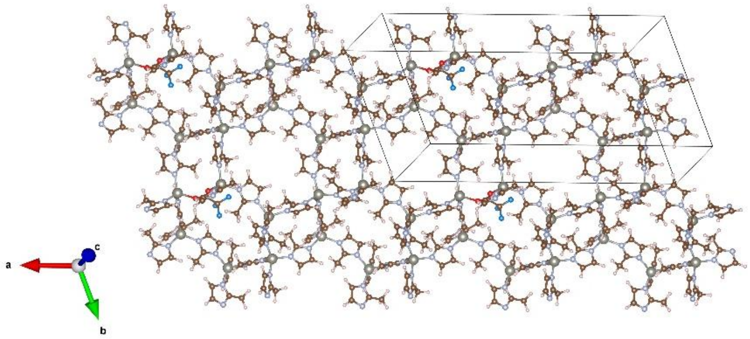

Figure S6. DFT analyzed bulk H-F-ZIF model containing four-unit cells.

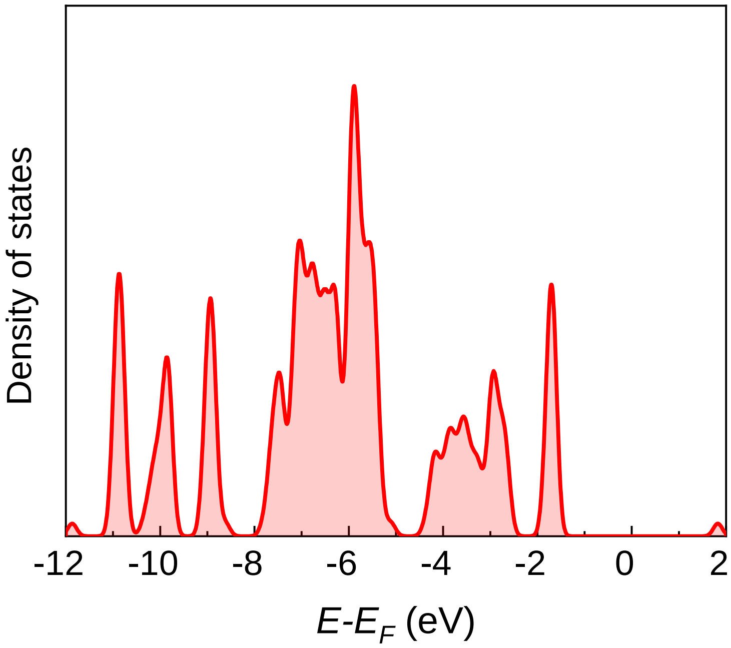

Figure S7. Total density of states of H-F-ZIF.

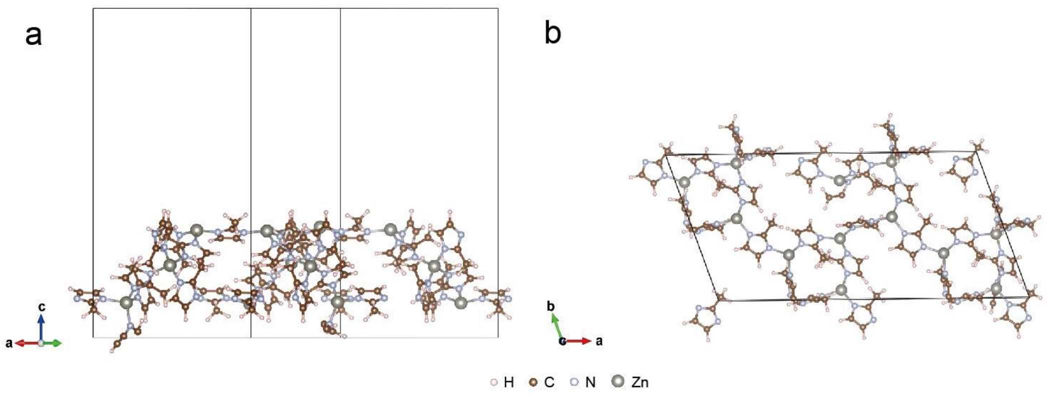

Figure S8. a) Side view and b) top view of DFT analyzed ZIF-8 surface model.

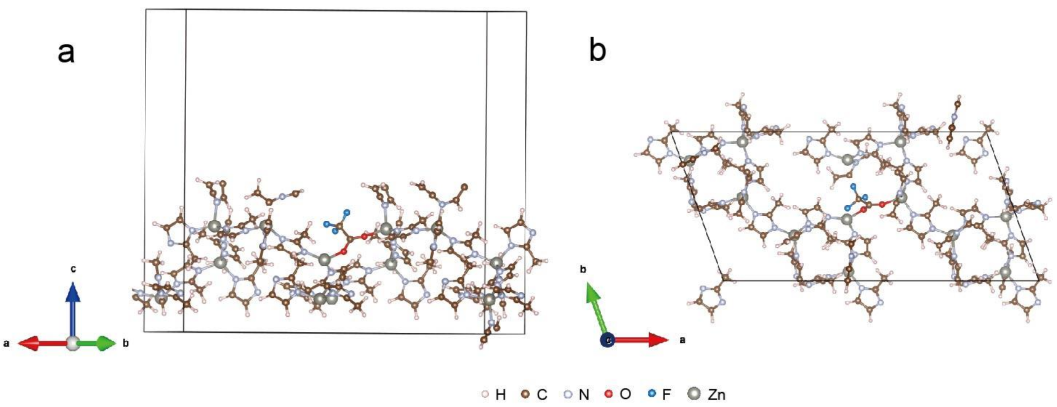

Figure S9. a) Side view and b) top view of DFT analyzed H-F-ZIF surface model.

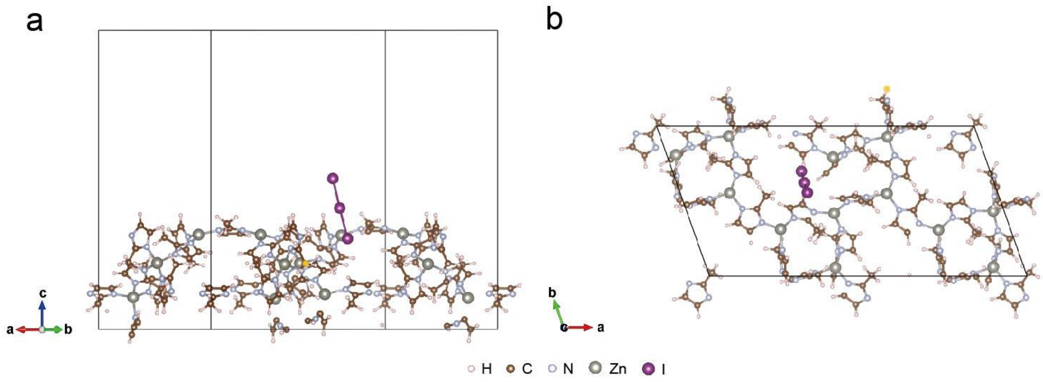

Figure S10. a) Side view and b) top view of DFT analyzed I_3^-- ZIF-8 surface model.

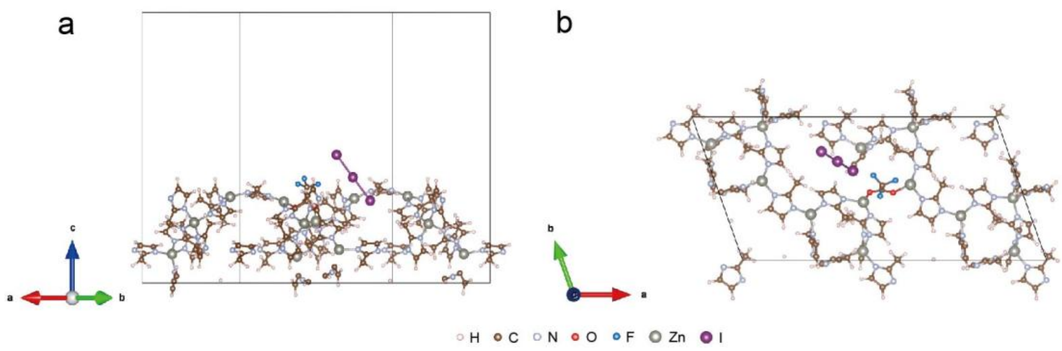

Figure S11. a) Side view and b) top view of DFT analyzed I_3^-- H-F-ZIF surface model.

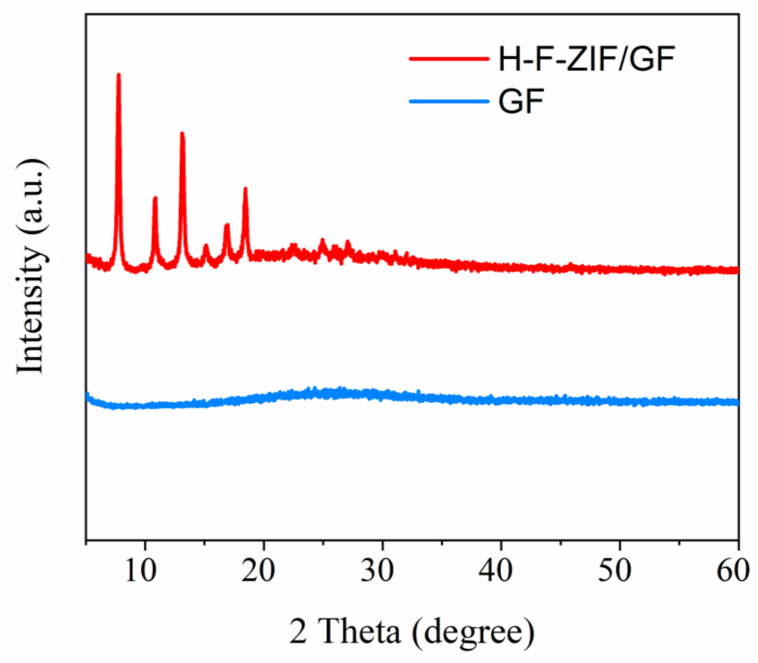

Figure S12. XRD patterns of GF and H-F-ZIF/GF.

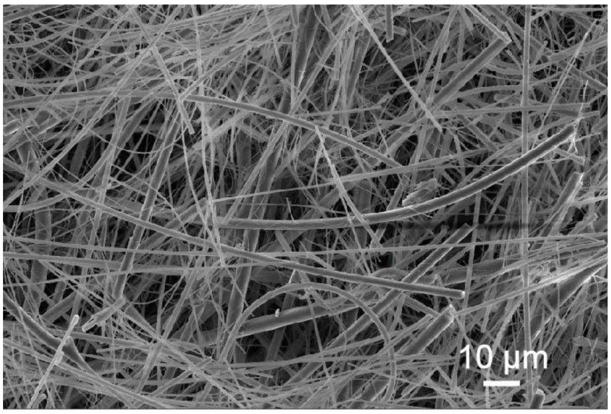

Figure S13. FESEM image of GF separator.

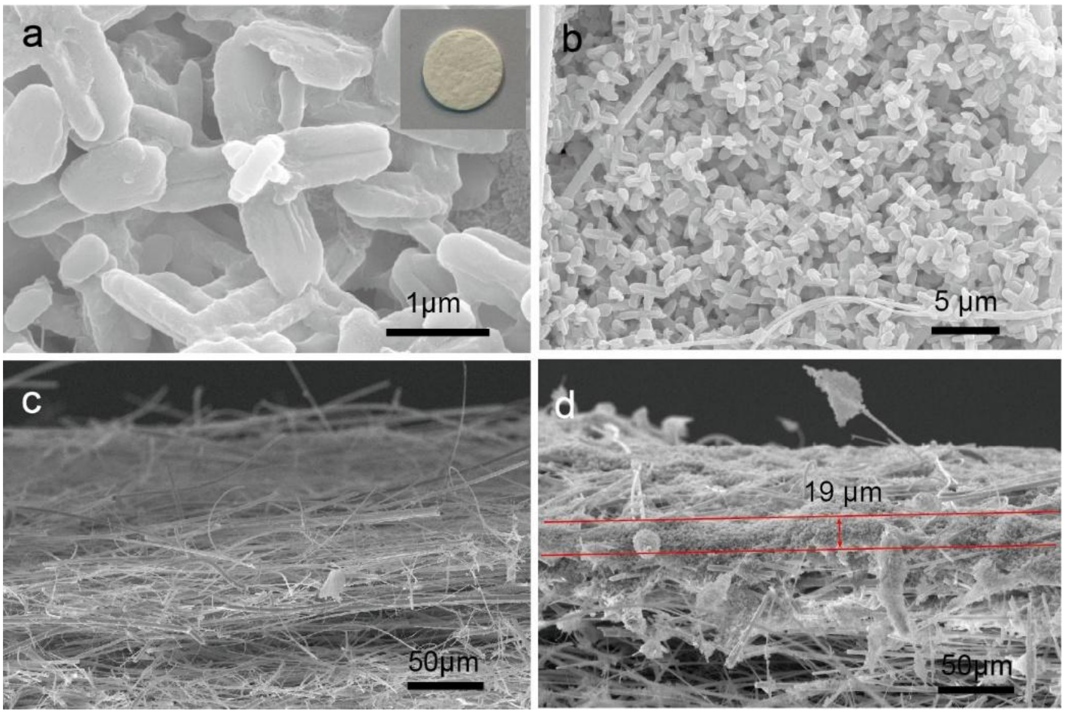

Figure S14. a, b) FESEM images of H-F-ZIF. Cross-sectional FESEM images of c) GF and d) H-F-ZIF/GF. The inset in (a) is a digital photo.

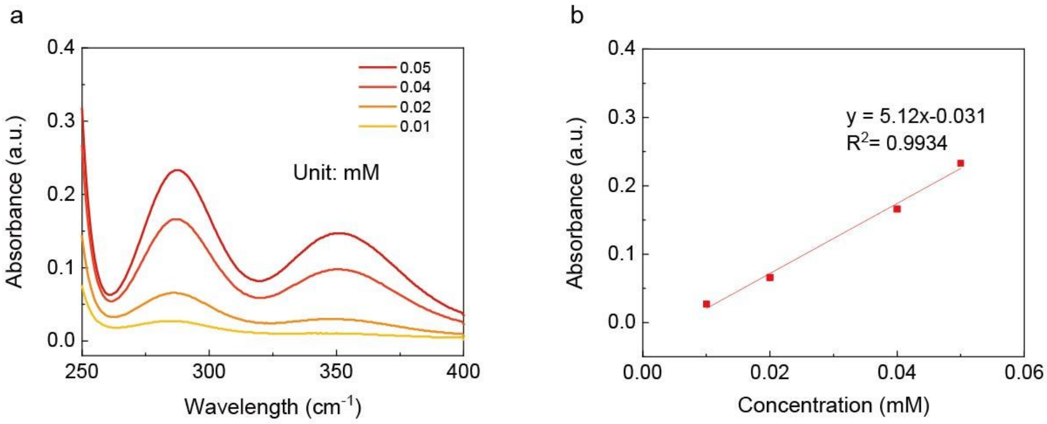

Figure S15. a) UV-vis spectra of triiodide solutions with different concentrations. b) Corresponding working plots revealing the relationship between concentration and absorbance.

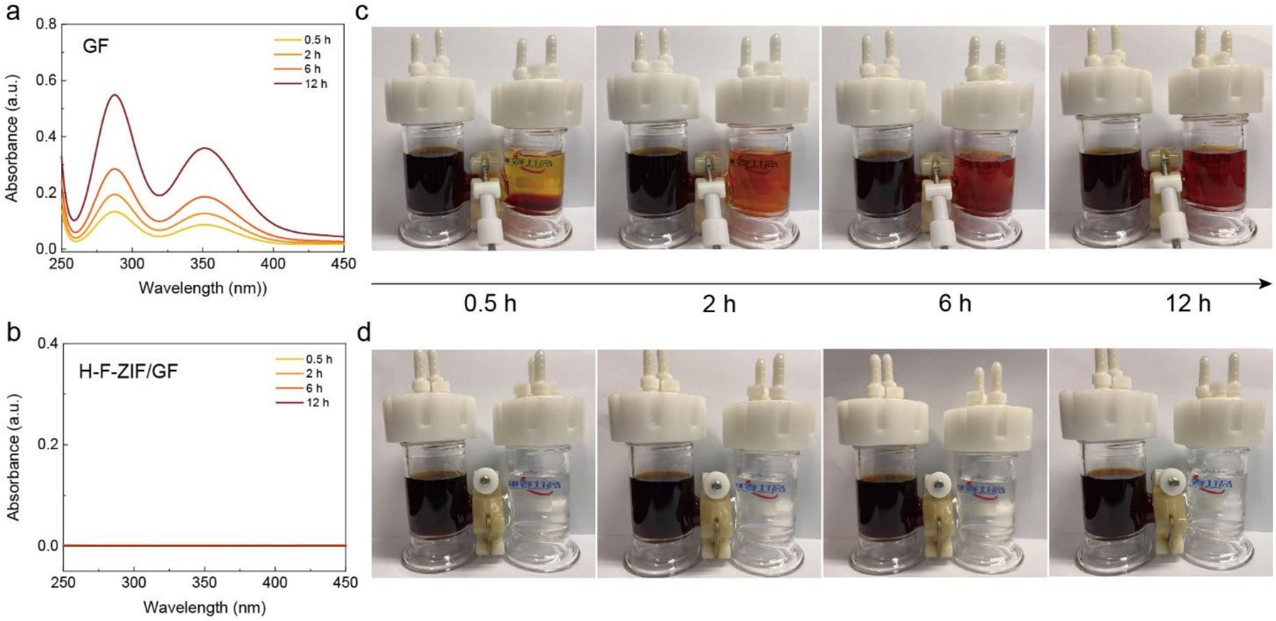

Figure S16. UV-vis spectra representing the change in I_3^- concentration over time in the right chambers of H-type cells using a) GF and b) H-F-ZIF/GF. Optical images of the permeation experiments of high concentration of I_3^- in the H-type cell assembled with c) GF and d) H-F-ZIF/GF.

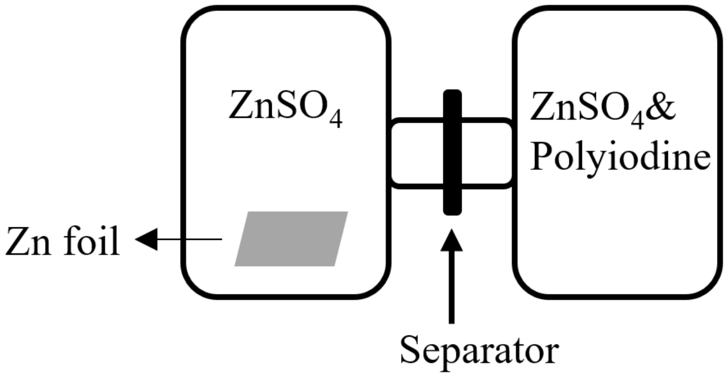

Figure S17. Schematic illustration of the experiment of the Zn foil immersed in the left chamber of an H-type cell.

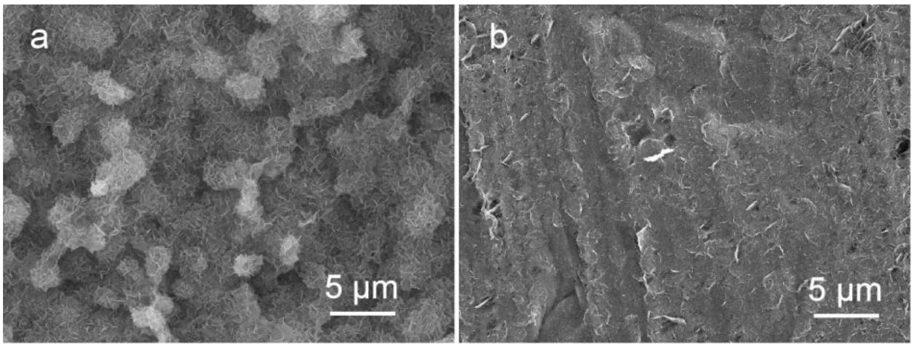

Figure S18. FESEM images of the Zn foil immersed in the left chamber of the H-type cell with a) GF and b) H-F-ZIF/GF.

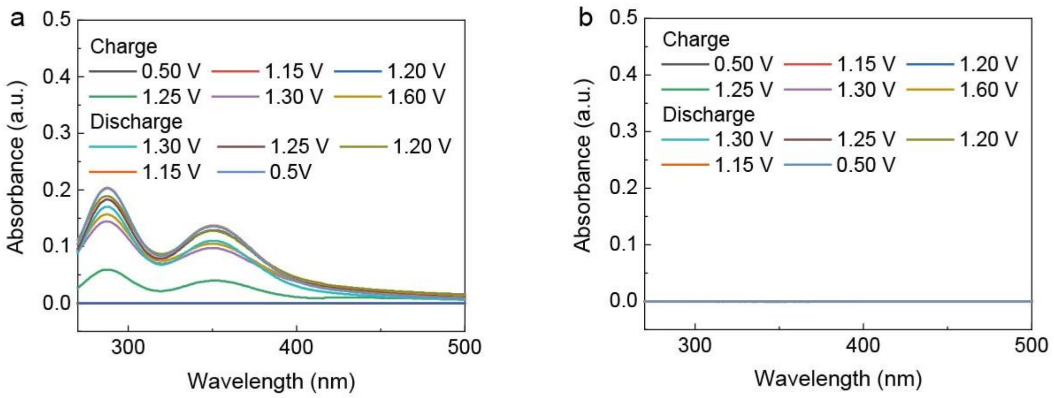

Figure S19. Ex situ UV-vis spectra of the solution in the left chamber during the charge-discharge process in the H type cell with a) GF and b) H-F-ZIF/GF separator.

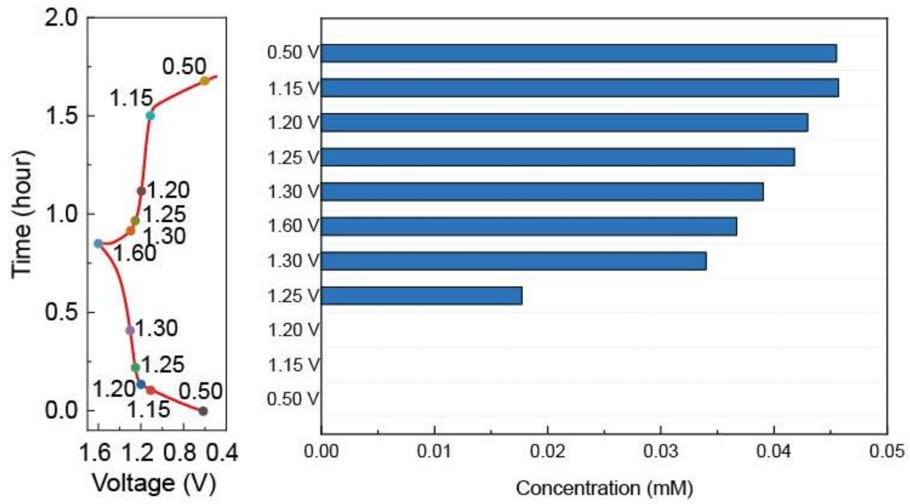

Figure S20. Voltage profile and corresponding calculated I_3^- concentration in H-type cell with GF separator during the charge-discharge process.

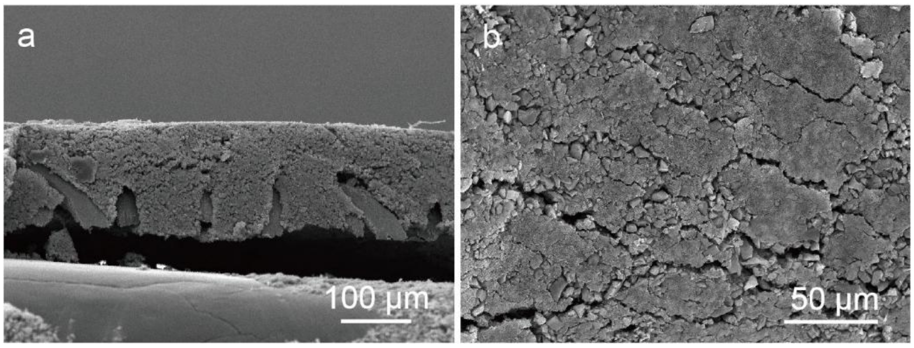

Figure S21. a) Cross-sectional and b) top view FESEM images of the AC cathode.

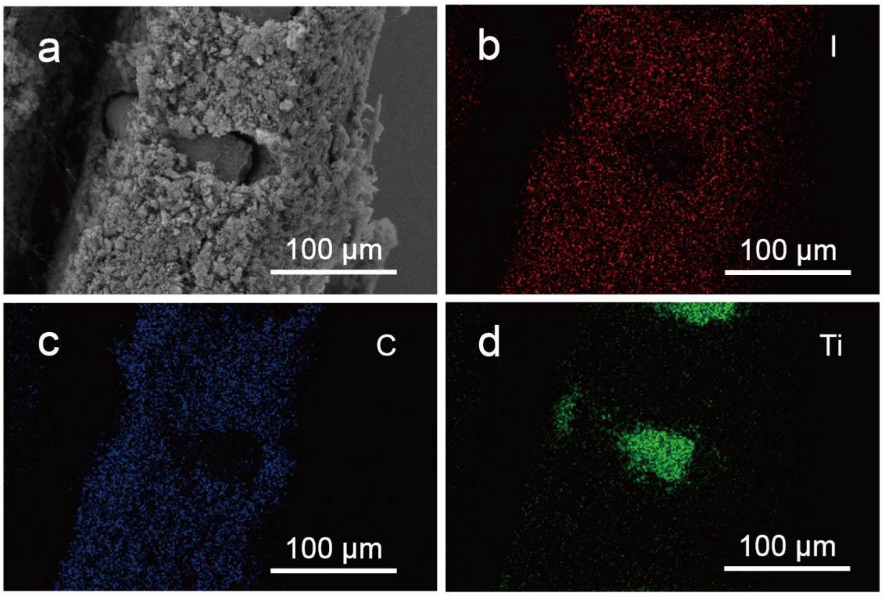

Figure S22. Cross-sectional elemental mapping images of cathode.

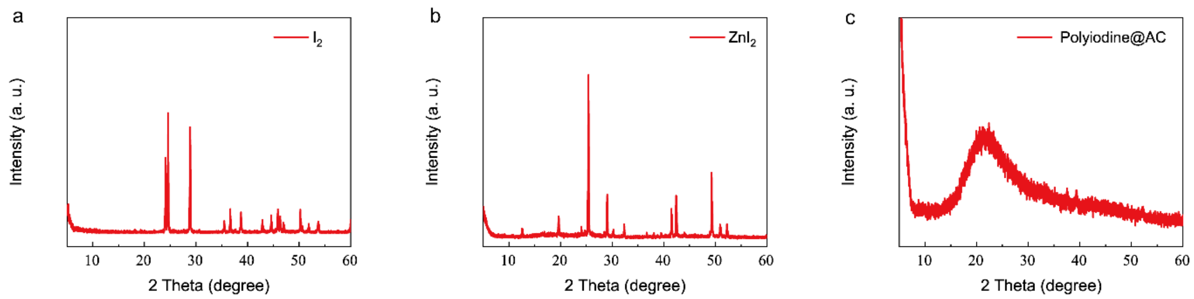

Figure S23. XRD patterns of a) I_2, b) ZnI_2, and c) polyiodide@AC.

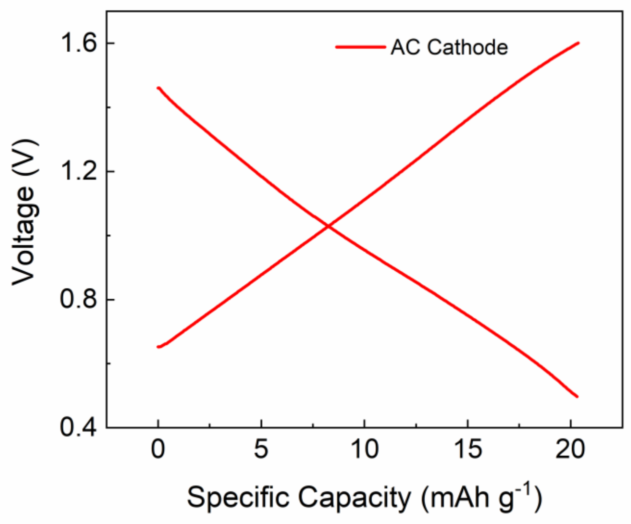

Figure S24. Voltage-capacity curves of the battery using blank AC cathode at a current density of 1.2 A g^{-1}.

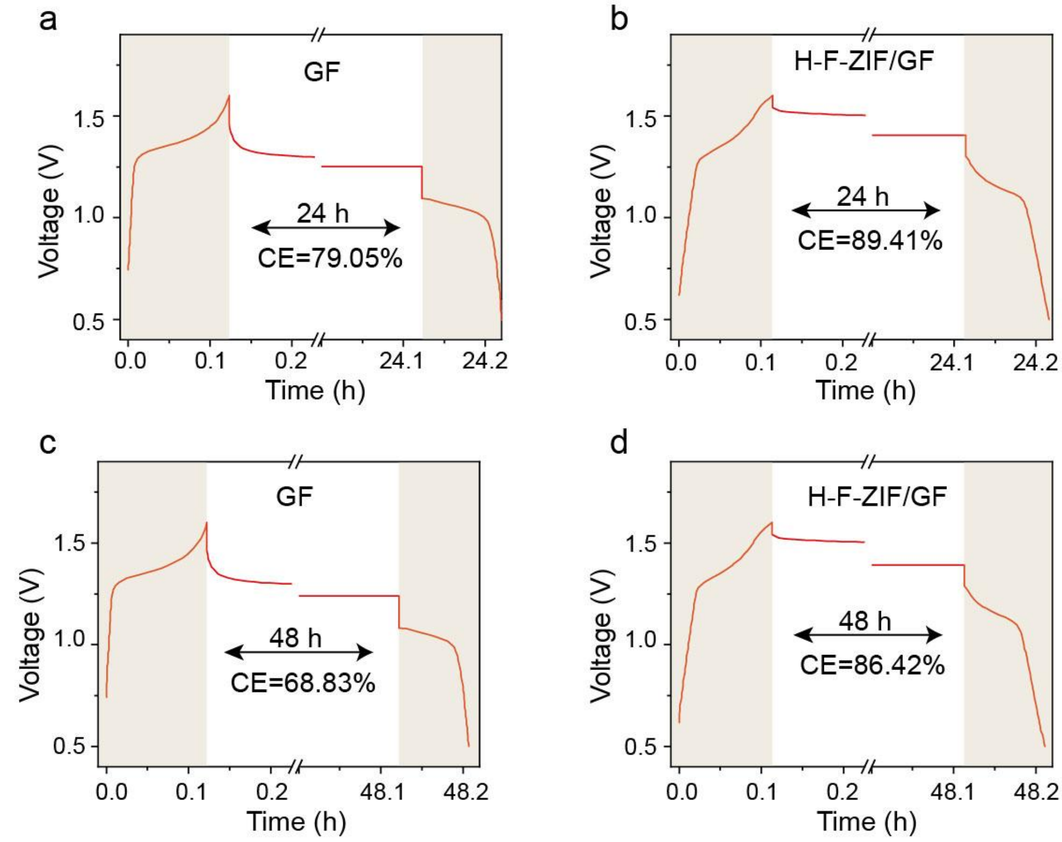

Figure S25. Self-discharge test curves of Zn-I_2 batteries using a, c) GF and b, d) H-F-ZIF/GF separators.

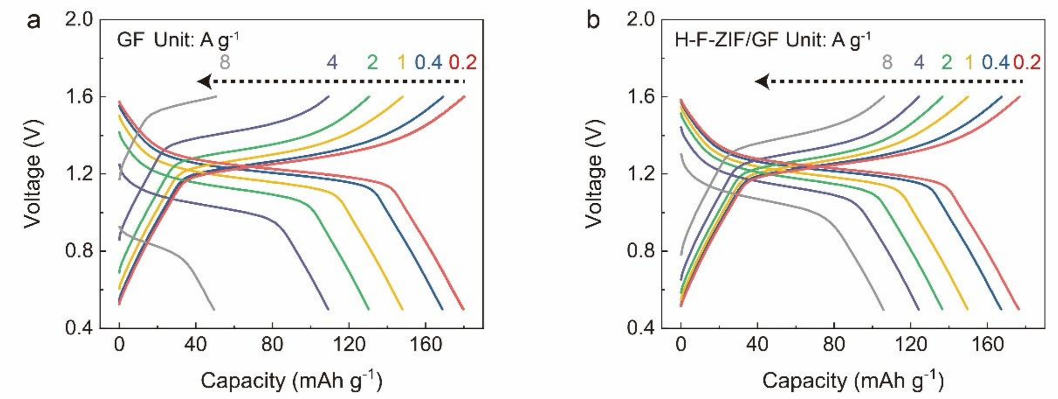

Figure S26. Voltage-capacity curves of Zn-I_2 full cells under different current densities with a) GF and b) H-F-ZIF/GF.

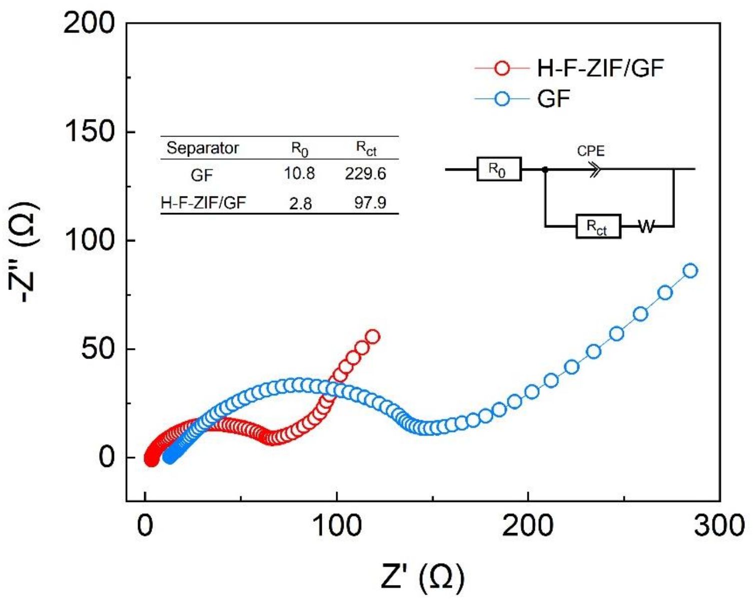

Figure S27. Nyquist plots of cycled Zn-I_2 batteries using GF and H-F-ZIF/GF. The inset shows the equivalent circuit and the fitted R_0 (ohmic resistance) and R_{ct} (charge transfer resistance).

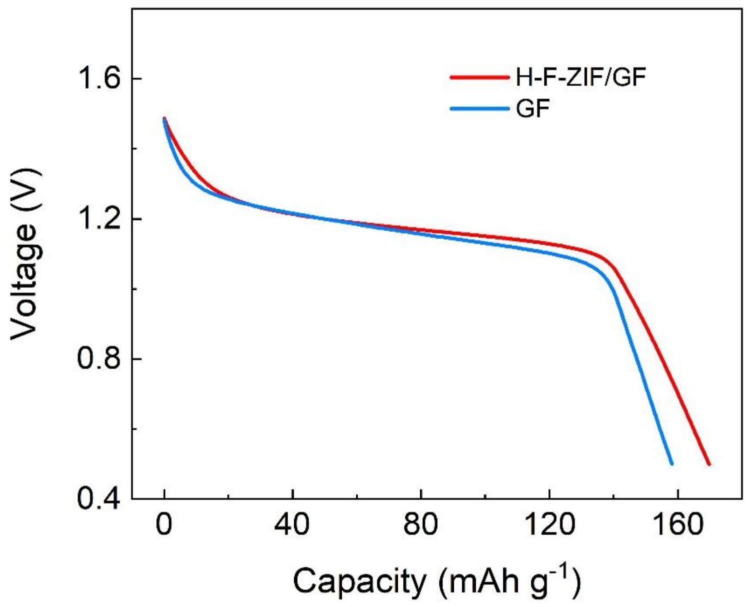

Figure S28. The discharge voltage profiles of Zn-I_2 batteries with H-F-ZIF/GF and GF separators after 200 cycles.

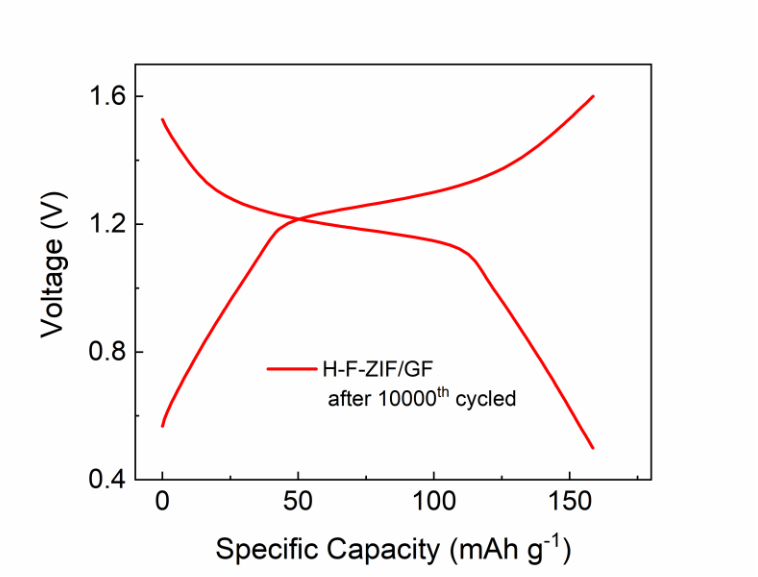

Figure S29. Charge-discharge voltage profiles of the Zn-I_2 battery with H-F-ZIF/GF at a current density of 1.2 A g^{-1} after 10000 cycles.

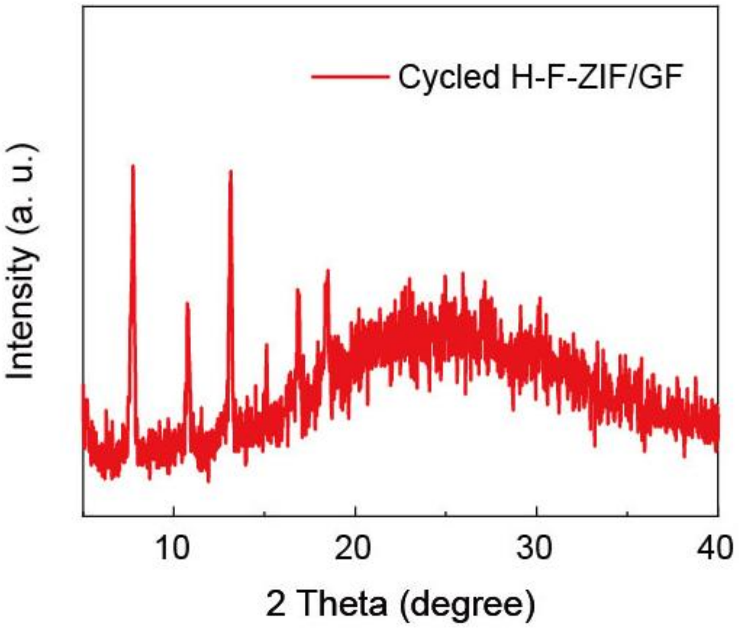

Figure S30. XRD pattern of cycled H-F-ZIF/GF.

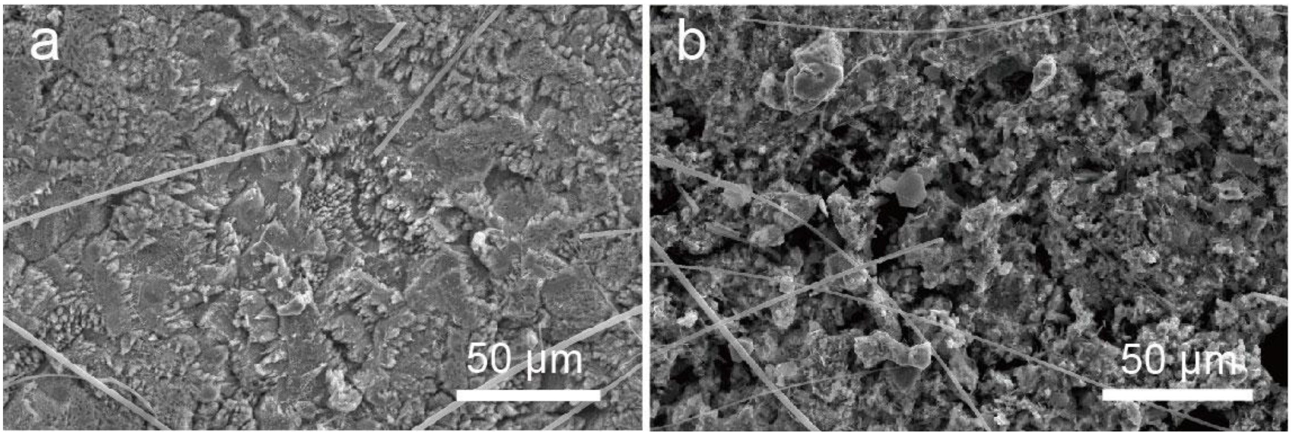

Figure S31. FESEM images of Zn anodes disassembled from the Zn-I_2 full cells after 300 cycles with a) H-F-ZIF, and b) GF.

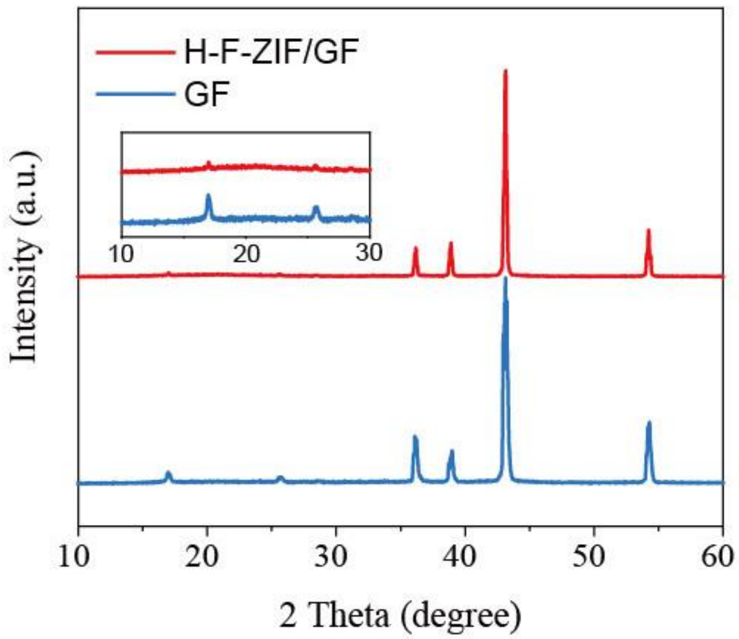

Figure S32. The XRD patterns of the cycled Zn anode in Zn-I_2 full batteries with GF and H-F-ZIF/GF.

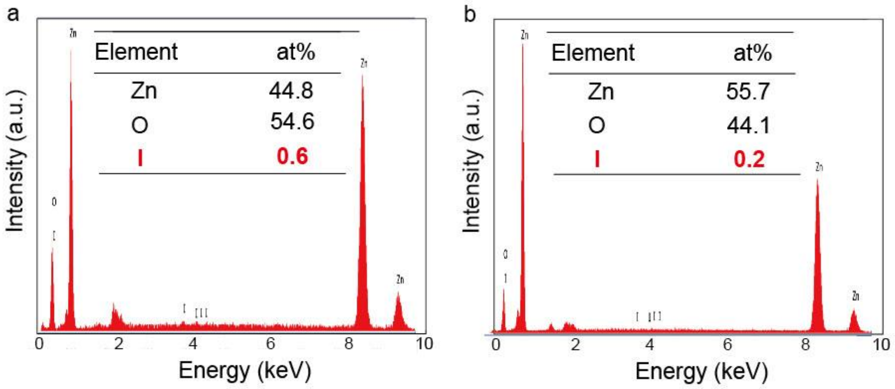

Figure S33. EDX spectra of cycled Zn anodes in Zn-I_2 full batteries with a) GF, and b) H-F-ZIF/GF.

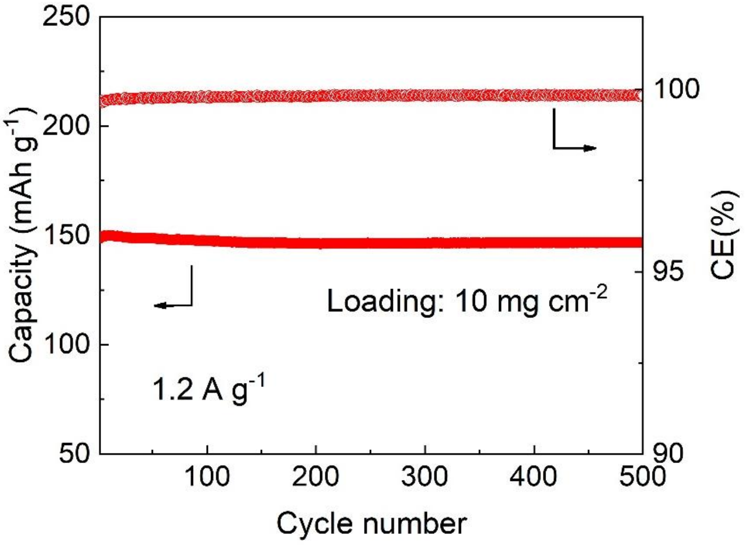

Figure S34. Cycling performance of a Zn-I_2 full cell using H-F-ZIF/GF with high iodine loading of 10 mg cm^{-2} at 1.2 A g^{-1}.

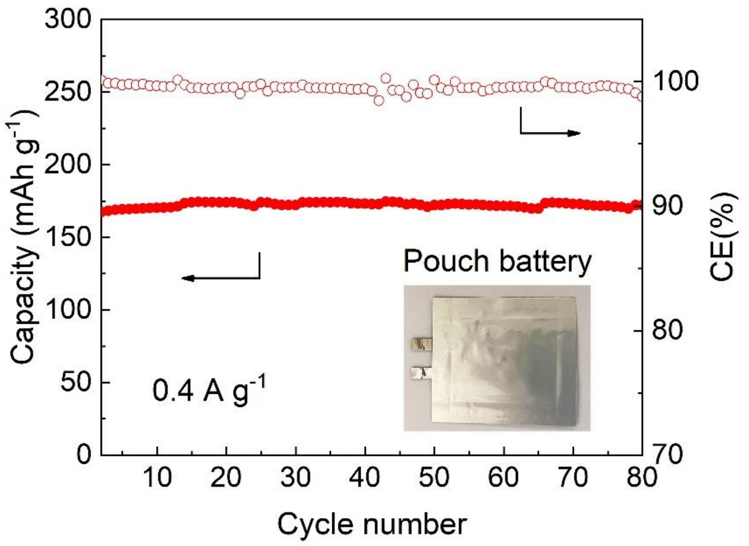

Figure S35. Cycling stability of a Zn–I_2 pouch cell using H-F-ZIF/GF separator at 0.4 A g^{-1}.

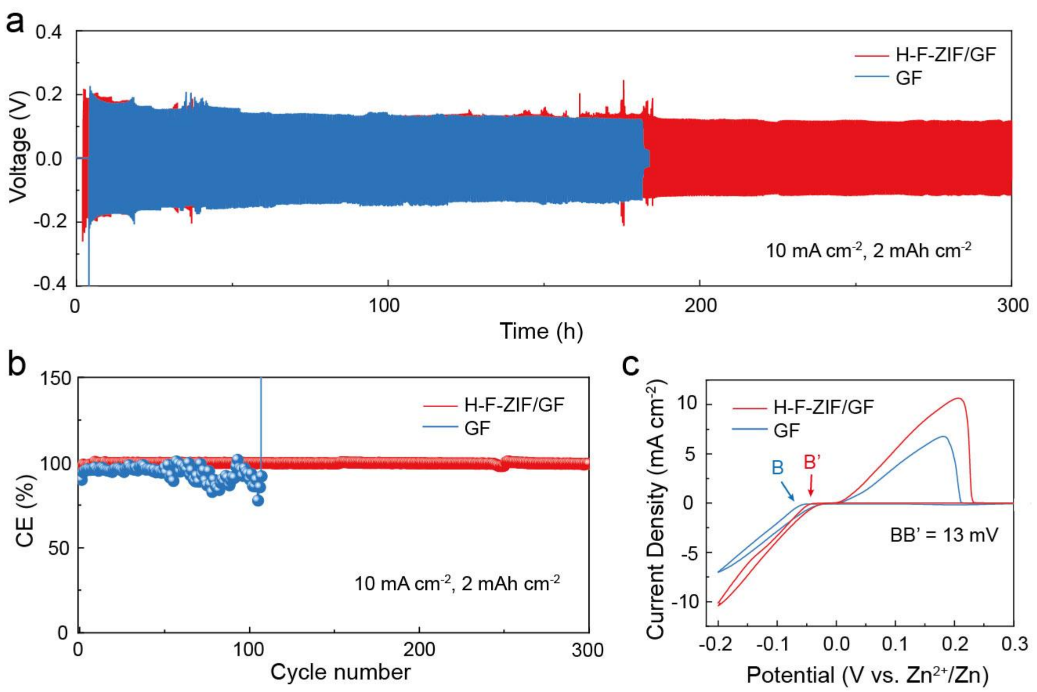

Figure S36. a) Cycling performance at 10 mA cm^{-2} for the Zn//Zn symmetric cells using different separators. b) CE plots of the Zn//Cu asymmetric cells tested at 10 mA cm^{-2} using different separators. c) CV curves of the asymmetric cells using different separators.

Supplementary References

[1] G. Kresse, J. Furthmuller, *Phys. Rev. B Condens Matter*. **1996**, 54, 11169.

[2] G. Henkelman, A. Arnaldsson, H. Jónsson, *Comput. M*ater. *Sci.* **2006**, 36, 354.

[3] G. Kresse, J. Furthmüller, *Comput. Mater. Sci.* **1996**, 6, 15.

[4] P. E. Blochl, *Phys. Rev. B Condens Matter.* **1994**, 50, 17953.

[5] G. Kresse, D. *Joubert, Phys. Rev. B* **1999**, 59, 1758.

[6] J. P. Perdew, K. Burke, M. Ernzerhof, *Phys. Rev. Lett.* **1996**, 77, 3865.
